# Childbirth care in Egypt: a repeat cross-sectional analysis using Demographic and Health Surveys between 1995 and 2014 examining use of care, provider mix and immediate postpartum care content

**DOI:** 10.1186/s12884-020-2730-8

**Published:** 2020-01-20

**Authors:** Miguel Pugliese-Garcia, Emma Radovich, Oona M. R. Campbell, Nevine Hassanein, Karima Khalil, Lenka Benova

**Affiliations:** 1 Faculty of Public Health and Policy, Faculty of Epidemiology and Population Health, London School of Hygiene and Tropical Medicine, Tavistock Place, London, WC1H 9SH UK; 2 Faculty of Epidemiology and Population Health, London School of Hygiene and Tropical Medicine, Keppel Street, London, WC1E 7HT UK; 3 Gynuity Health Projects, Egypt team, 220 East 42nd, New York, NY 10017 USA; 4 Oxford Policy Management, New Delhi, India; 5 Institute of Tropical Medicine, Antwerp, Belgium

**Keywords:** Childbirth care, Delivery care, Egypt, Quality, Demographic and health survey, Caesarean section, Postpartum care, Breastfeeding initiation, Early discharge, Length of stay

## Abstract

**Background:**

Egypt has achieved important reductions in maternal and neonatal mortality and experienced increases in the proportion of births attended by skilled professionals. However, substandard care has been highlighted as one of the avoidable causes behind persisting maternal deaths. This paper describes changes over time in the use of childbirth care in Egypt, focusing on location and sector of provision (public versus private) and the content of immediate postpartum care.

**Methods:**

We used five Demographic and Health Surveys conducted in Egypt between 1995 and 2014 to explore national and regional trends in childbirth care. To assess content of care in 2014, we calculated the caesarean section rate and the percentage of women delivering in a facility who reported receiving four components of immediate postpartum care for themselves and their newborn.

**Results:**

Between 1995 and 2014, the percentage of women delivering in health facilities increased from 35 to 87% and women delivering with a skilled birth attendant from 49 to 92%. The percentage of women delivering in a private facility nearly quadrupled from 16 to 63%. In 2010–2014, fewer than 2% of women delivering in public or private facilities received all four immediate postpartum care components measured.

**Conclusions:**

Egypt achieved large increases in the percentage of women delivering in facilities and with skilled birth attendants. However, most women and newborns did not receive essential elements of high quality immediate postpartum care. The large shift to private facilities may highlight failures of public providers to meet women’s expectations. Additionally, the content (quality) of childbirth care needs to improve in both sectors. Immediate action is required to understand and address the drivers of poor quality, including insufficient resources, perverse incentives, poor compliance and enforcement of existing standards, and providers’ behaviours moving between private and public sectors. Otherwise, Egypt risks undermining the benefits of high coverage because of substandard quality childbirth care.

## Background

Although substantial progress was made to meet the Millennium Development Goals, maternal and perinatal mortality remain unacceptably high in most low- and middle-income countries (LMICs). Globally, the largest burden of maternal deaths occurs during labor, delivery and the immediate postnatal period, namely the peripartum period [1], and similar patterns hold for babies. The preventive and curative interventions that improve maternal and perinatal survival are known and supported by rigorous evidence. It was thought that improving access to and utilisation of facility-based childbirth care had an important role to play in reducing preventable maternal and perinatal deaths [2, 3]. However, increasing evidence highlights that poor quality facility-based care challenges this assumption [4–6].

Egypt’s population is estimated to have reached 99 million people [7] and, after years of decline, its total fertility rate increased from 3.0 in 2008 to 3.5 in 2014 [8]. Despite potential reductions in the fertility rate more recently [9], the country’s health system has had to provide care for an increasing number of births. For example, there were an estimated 2.7 million births in 2014, a 46% increase from 1.9 million in 2006 [10]. Although Egypt’s maternal mortality ratio decreased from 174 per 100,000 live births in 1992 to 37 in 2017 [11, 12], and its neonatal mortality rate from 31 per 1000 live births in 1992 to 12 in 2017 [13], around 960 maternal and 30,000 neonatal deaths occur annually. Thanks to a high density population concentrated along the banks of the river Nile and in the Nile delta and the development of a large network of health facilities, distance and lack of transport were rarely identified as avoidable factors for maternal death in Egypt [11, 14, 15]. In contrast, substandard care by the obstetric team, absence or poor quality of antenatal care, and delays in recognising problems and seeking care were identified as the most important avoidable causes of maternal death [14, 16]. Moreover, large socio-economic disparities in the utilisation of facility-based childbirth care continue to exist [17].

Healthcare in Egypt is provided by a broad spectrum of modern and traditional health care providers, ranging from governmental, parastatal, university, military, for-profit, non-governmental organizations (NGOs), and traditional practitioners, with variable quality and cost [18, 19]. The Ministry of Health and Population (MOHP) is a major provider of primary, preventive and curative care, and has an oversight responsibility (but limited authority and capacity) to regulate private providers. During the period between 2007 and 2016, Egypt had 8 physicians and 14 nurses and midwives per 10,000 population [20]. A high proportion of health expenditure - 62% in 2016 - came from out-of-pocket payments [21], with only 8.1% of women aged 15 to 49 years covered by some form of public or private health insurance in 2014 [22]. Childbirth care in public hospitals is provided under three pricing schemes – public, *iqtisady* (for patients enrolled with the health insurance organization, the HIO), and *fondoqy* (“hotel” or private service, which enables obstetricians to admit and attend private-practice patients).

In 2014, the percentage of women delivering with a skilled birth attendant (SBA) reached 92%, with 87% of women in the country delivering in a facility [22]. However, there is limited evidence on which sector is providing childbirth care, on quality of care, or on which women are being left behind in terms of coverage and quality. These aspects are crucial to understanding the status of childbirth care in Egypt and developing policies that will further improve the well-being of its women and children.

The objective of this paper is to describe the changes over time in the use of childbirth care in Egypt, focusing on the sector of provision (public versus private), and the content of this care, nationally and by region.

## Methods

### Data and study population

We used the five most recent Demographic and Health Surveys (DHS) conducted in Egypt in 1995, 2000, 2005, 2008, and 2014. In analyses, we refer to the surveys using the span of their five-year recall periods for which details on women’s live births were collected (i.e., 1995 survey: 1991–1995). DHS are cross-sectional nationally representative household surveys with a multistage cluster sampling strategy. Their model questionnaires are adapted to each country’s circumstances and include questions on household and individual characteristics, fertility and family planning, maternal and child health and details on antenatal and childbirth care. All ever-married women aged 15–49 years with a live birth in the surveys’ five-year recall period were included in the analysis. We examined women’s self-report of childbirth care source and the components of immediate postpartum care for the most recent live birth.

### Subnational regions

**Fig. 1:**
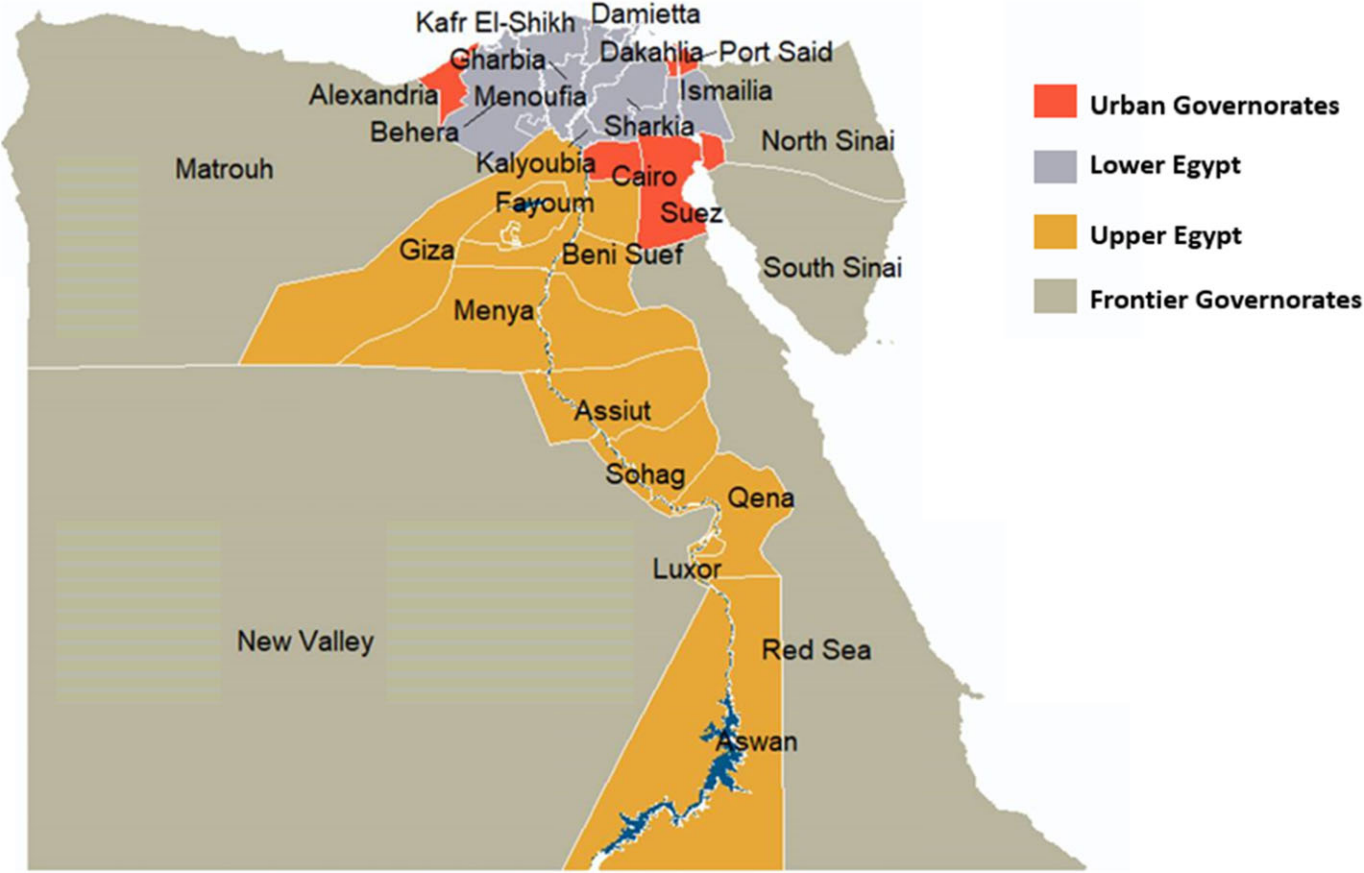
Map of Egypt’s governorates as classified in the Demographic and Health Surveys adapted using data from the Humanitarian Data Exchange (HDX) [23] under the CC BY-IGO license

All five DHS used the same six major administrative regionsto produce subnational estimates: 1) Urban Governorates (four cities without rural populations: Cairo, Alexandria, Port Said, Suez), 2) urban Lower Egypt (ULE), 3) rural Lower Egypt (RLE), 4) urban Upper Egypt (UUE), 5) rural Upper Egypt (RUE), and 6) Frontier Governorates (Red Sea, New Valley, Matrouh, North Sinai and South Sinai governorates). Figure 1 depicts the 27 Egyptian governorates organised according to the DHS’ classification. In 2014, North and South Sinai were not surveyed for security reasons. While national estimates were not affected by this exclusion because only a small percentage of the total population (< 1%) resided there, the 2014 Frontier Governorates estimates should be compared to previous surveys with caution.

### Definitions and analysis

Details on the indicators and definitions used in this study can be found in Additional file 1. In our analysis, we considered women to be in need of childbirth care if they had a live birth in the survey recall period [24]. Women were considered to have had a facility delivery if they reported giving birth in any health facility, and assisted by an SBA if they reported being assisted by a doctor, nurse or midwife. To assess the content of childbirth care, we used four components of immediate postpartum care as proxies. These included: 1) initiating breastfeeding within an hour of birth; 2) weighing the baby; 3) checking on the woman’s health while still in the facility; 4) reporting a minimally acceptable length-of-stay in the facility. We assessed the percentage of women reporting receiving each component and all components.

### Analysis

We first calculated the percentage of women delivering in any health facility, with a SBA and in private facilities. Then, we assessed the percentage of women delivering in facilities by sector (public and private), in each region and by household wealth. We estimated the percentage of women receiving each of four components of immediate postpartum care and all four components, by sector of provision, type of residence, household wealth, mode of delivery (vaginal or caesarean (C)-section), and region. Data analysis was conducted in Stata SEv14 (College Station, TX), using the *svyset* command to account for survey design of each survey (sample weights, clustering and stratification). We used tabulations and Chi-square tests to provide descriptive statistics and assess the statistical significance of different distributions. We produced estimates for each survey separately (not pooled).

## Results

### Study population

**Fig. 2:**
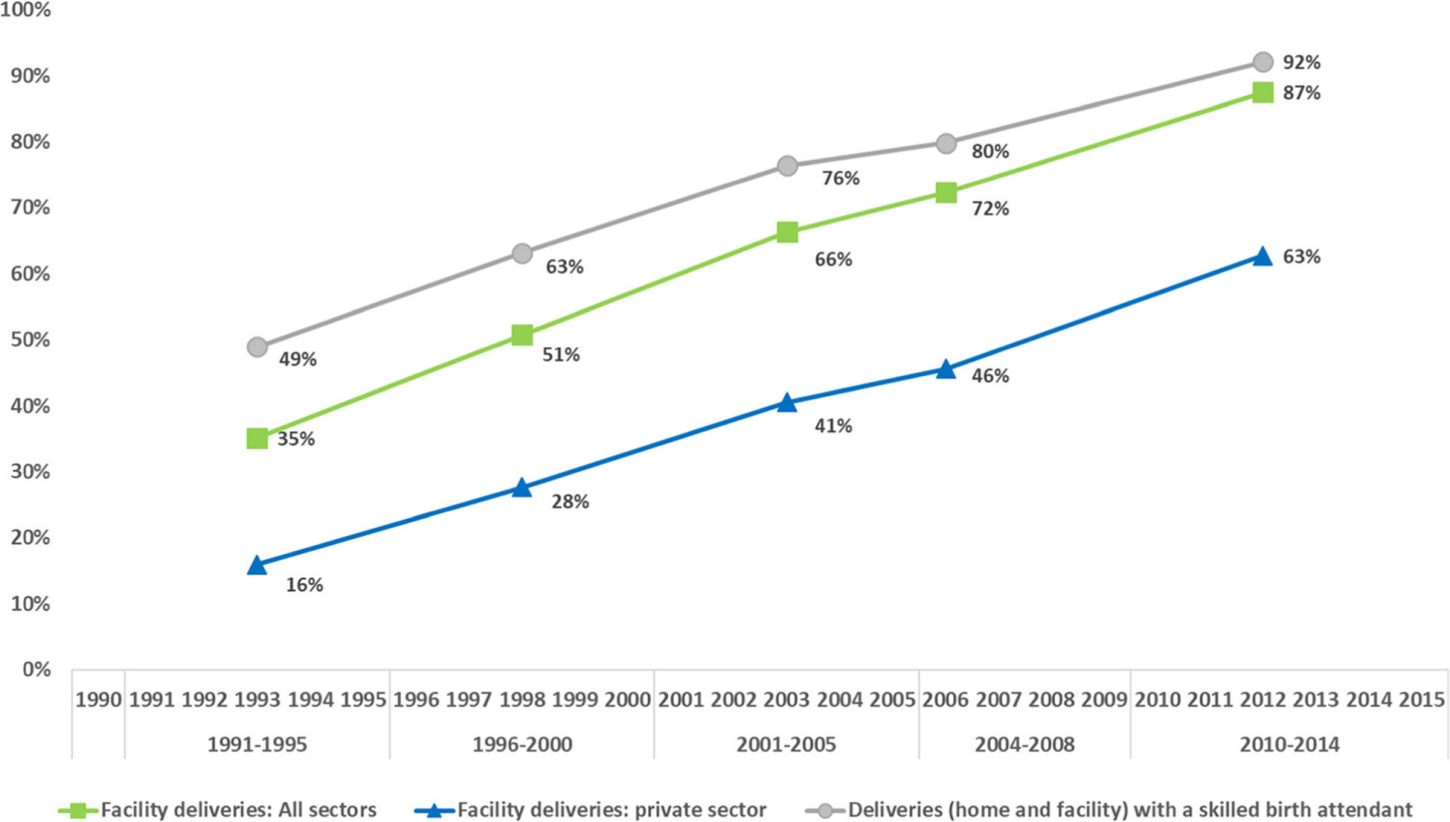
Percentage of women in need of childbirth care delivering in a facility, delivering in a private facility and delivering assisted by an SBA between 1991 and 1995 and 2010–2014

**Fig. 3:**
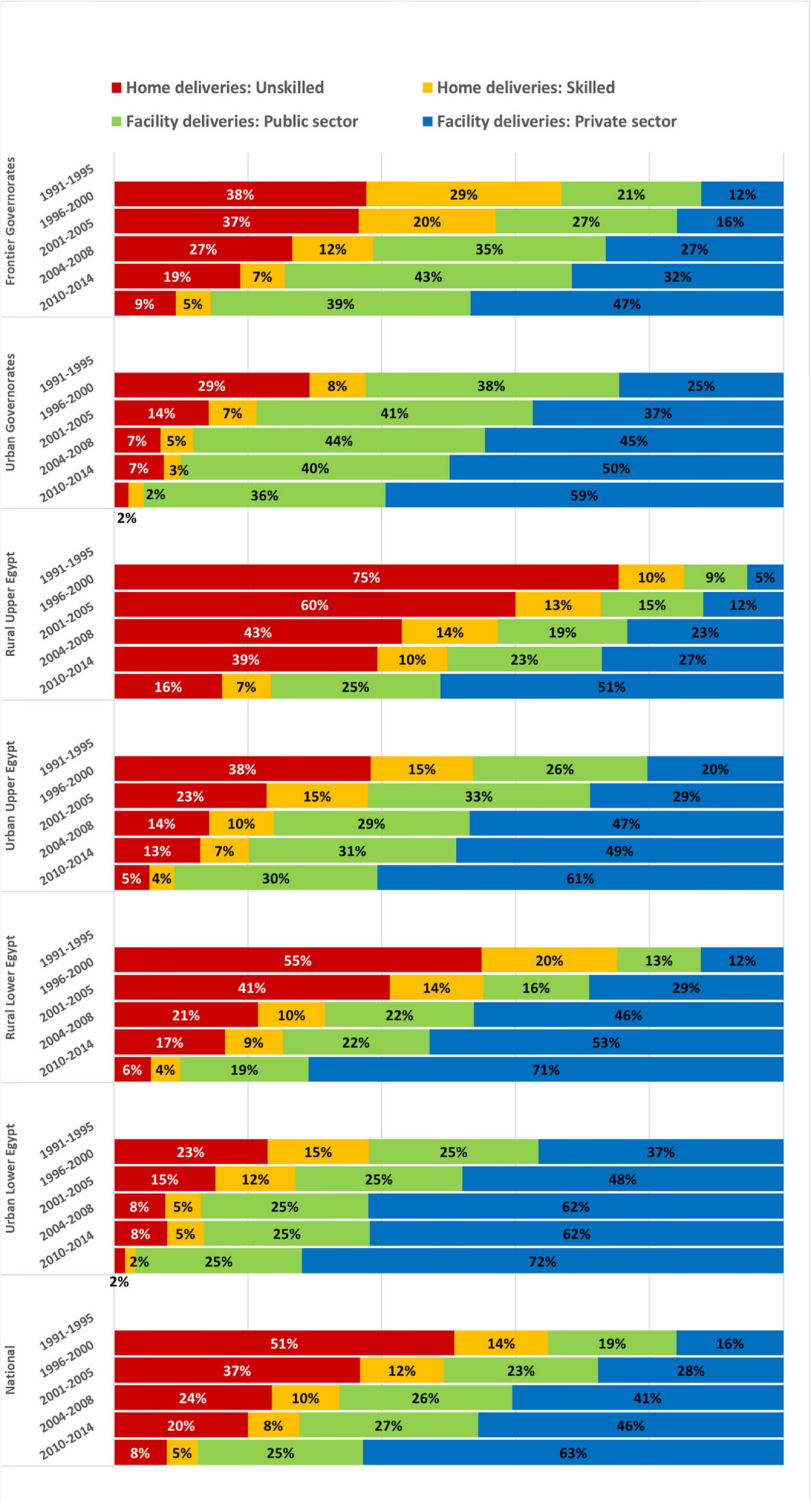
Percentage of women in need of childbirth care between 1991 and 1995 and 2010–2014, by location, attendant and sector, by region

**Fig. 4:**
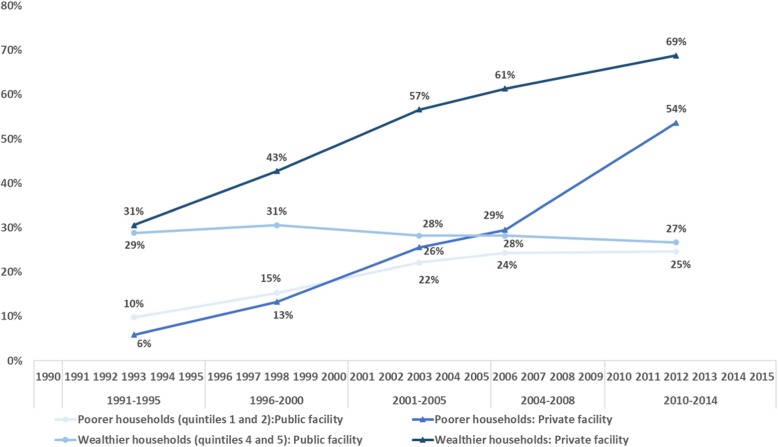
Percentage of women in need of childbirth care that delivered in a public or a private facility, stratified by household wealth

In total, data on childbirth care use and key variables of interest were available for 45,387 women in need of care across the five surveys, of whom 28,968 delivered in a health facility. The characteristics of the study participants, stratified by survey year, are shown in Additional file 2, while percentages, 95% confidence intervals and *p*-values of the data used in Figs. 2, 3 and 4 can be found in Additional file 3.

### National trends in total and private childbirth care use between 1995 and 2014

Between 1991 and 1995 and 2010–2014, the percentage of all women in need of childbirth care who delivered in a facility increased from 35 to 87%; the percentage who delivered in private sector facilities over the same time periods increased from 16 to 63% (Fig. 2) and the percentage of women assisted by an SBA increased from 49 to 92%. On all five surveys, over 99% of women delivering in facilities reported being attended by a doctor. Among women who gave birth at home, the percentage being attended by an SBA increased from 22 to 37% over the study period. Among women delivering at home with an SBA in 2010–2014, 37% were attended by a doctor and 63% by a nurse or midwife.

### National and regional change in facility-based childbirth and type of provider between 1995 and 2014

The percentage of women in need of childbirth care who delivered at home (regardless of birth attendant type) decreased in all regions between 1991 and 1995 and 2010–2014 (Fig. 3). We observed the largest absolute percentage reductions in Rural Lower Egypt (RLE) and Rural Upper Egypt (RUE): 65 and 62 percentage points (pp), respectively. Among all women in need of childbirth care, the percentage giving birth in public facilities increased in RLE (6 pp), RUE (16 pp), Urban Upper Egypt (UUE) (16 pp) and Frontier governorates (18 pp) and decreased in Urban governorates (2 pp) and Urban Lower Egypt (ULE) (1 pp). The share of childbirth care occurring in private facilities increased in all regions. The largest increases were observed in RLE (59 pp) and RUE (46 pp).

### Time trends in the use of public and private facility-based childbirth care by wealth quintile

Among women in need of childbirth care from the two poorest quintiles, the percentage delivering in a public facility increased from 8% in 1991–1995 to 25% in 2010–2014, while the percentage delivering in a private facility rose from 5 to 51% (Fig. 4). Among women from the two wealthiest quintiles, 29% of women reported delivering in public facilities in 1991–1995, which remained virtually unchanged at 27% in 2010–2014, while the percentage who reported delivering in a private facility increased from 31% in 1005 to 69% in 2010–2014.

### Time trends and sector differences in type of birth

The percentage of women delivering by a C-section increased from 7% of all live births in 1991–1995 to 54% in 2010–2014, and from 20% of facility births to 62% during the same time periods (Table 1). Among women delivering in public facilities, the percentage delivering by C-section increased from 19 to 49%, compared to 22 and 67% of women delivering in private facilities. For women from the poorest 40% of households, the percentage of all births by C-section increased from 3 to 43%. For the wealthiest 40%, it increased from 13 to 64%.

**Table 1:** Percentage of livebirths by C-section between 1991 and 1995 and 2010–2014 across women delivering in a facility, all women and type of provider and household wealth

	1991–1995	1996–2000	2001–2005	2004–2008	2010–2014
Delivery location					
Facility births	20%	22%	32%	40%	62%
Sector					
Public facilities	19%	20%	29%	35%	49%
Private facilities	22%	23%	34%	43%	67%
Household wealth					
Poorer	3%	4%	12%	18%	43%
Wealthier	13%	18%	32%	41%	64%
All livebirths	7%	11%	21%	29%	54%

### Differences in the components of immediate postpartum care between public and private facilities

Table 2 shows the four components of immediate postpartum care measured in the 2014 DHS. Of these, having the minimum acceptable length of stay was the least commonly reported care component in both public and private facilities (14 and 5%, respectively, *p*-value< 0.001). This was followed by immediate initiation of breastfeeding (29 and 24%, *p*-value< 0.001). In contrast, being checked before discharge was almost universal (89 and 92%, *p*-value< 0.001). In total, 2% of women received all four components in public facilities, compared to 1% of women in private facilities (*p*-value = 0.001).

**Table 2:** Percentage of women delivering in public and private facilities reporting receiving each component of immediate postpartum care and all components, by type of residence, household wealth and type of delivery on the 2014 Egypt DHS

	Baby breastfed in < 1 h			Baby weighed			Mother checked before discharge			Minimum acceptable lentgh of stay			All four components		
	Public	Private	*p*-value	Public	Private	*p*-value	Public	Private	*p*-value	Public	Private	*p*-value	Public	Private	*p*-value
Type of residence															
Urban	26%	23%	0.121	68%	75%	0.002	93%	95%	0.029	14%	6%	< 0.001	2%	1%	0.213
Rural	31%	25%	< 0.001	57%	66%	< 0.001	87%	91%	< 0.001	14%	5%	< 0.001	3%	1%	< 0.001
Household wealth															
Poorer	30%	23%	0.002	52%	59%	0.002	85%	89%	0.002	16%	5%	< 0.001	2%	1%	< 0.001
Wealthier	26%	23%	0.127	71%	75%	0.017	93%	95%	0.019	13%	6%	< 0.001	2%	1%	0.090
Type of delivery															
Vaginal	40%	36%	0.045	55%	63%	< 0.001	84%	85%	0.703	18%	11%	< 0.001	4%	2%	0.009
C-section	17%	19%	0.395	69%	71%	0.067	95%	96%	0.284	9%	2%	< 0.001	1%	0%	0.001
All women	29%	24%	< 0.001	62%	69%	< 0.001	89%	92%	< 0.001	14%	5%	< 0.001	2%	1%	< 0.001

Looking at differences by residence, we found that in urban areas, 68% of women delivering in public facilities reported their babies were weighed, compared to 75% in private (*p*-value = 0.002). In urban areas, 14% of women delivering in public facilities reported an acceptable length of stay, compared to 6% in private (*p*-value< 0.001). In rural areas, women delivering in public facilities reported their babies weighed less commonly (57%) than in private (66%, *p*-value< 0.001). A higher percentage of rural women receiving childbirth care reported breastfeeding within an hour of birth (31% public, 25% private, *p*-value< 0.001) and an acceptable length of stay (14% public, 5% private, *p*-value< 0.001).

Among users of public facilities, poorer women more commonly reported their babies were breastfed within an hour of birth (30%) compared to wealthier women (23%, *p*-value = 0.002). They were more likely to report a minimally acceptable length of stay (16%, wealthier women: 5%, *p*-value< 0.001). In contrast, poorer women delivering in private facilities more often reported their babies were weighed (59%) than wealthier women (52%, *p*-value = 0.002). Among women delivering in private facilities, wealthier women were more likely to report a minimum acceptable length of stay (13%) compared to poorest women (6%, *p*-value< 0.001).

Among women with vaginal births, those delivering in the public sector were more likely to have breastfed their newborn within an hour of birth (40%) compared to 36% in the private sector (*p*-value = 0.045) and to have had an acceptable length of stay (18% in public and 11% in private, *p*-value< 0.001). For having their baby weighted, the reverse pattern was seen: lower in public (55%) compared to private sector (63%). The sectors did not differ in the percentage of women checked before discharge.

Among women with a C-section, the content of care did not differ between the sectors except for the percentage reporting an acceptable length of stay, which was higher in public facilities (9%) than in private (2%, *p*-value< 0.001).

**Fig. 5:**
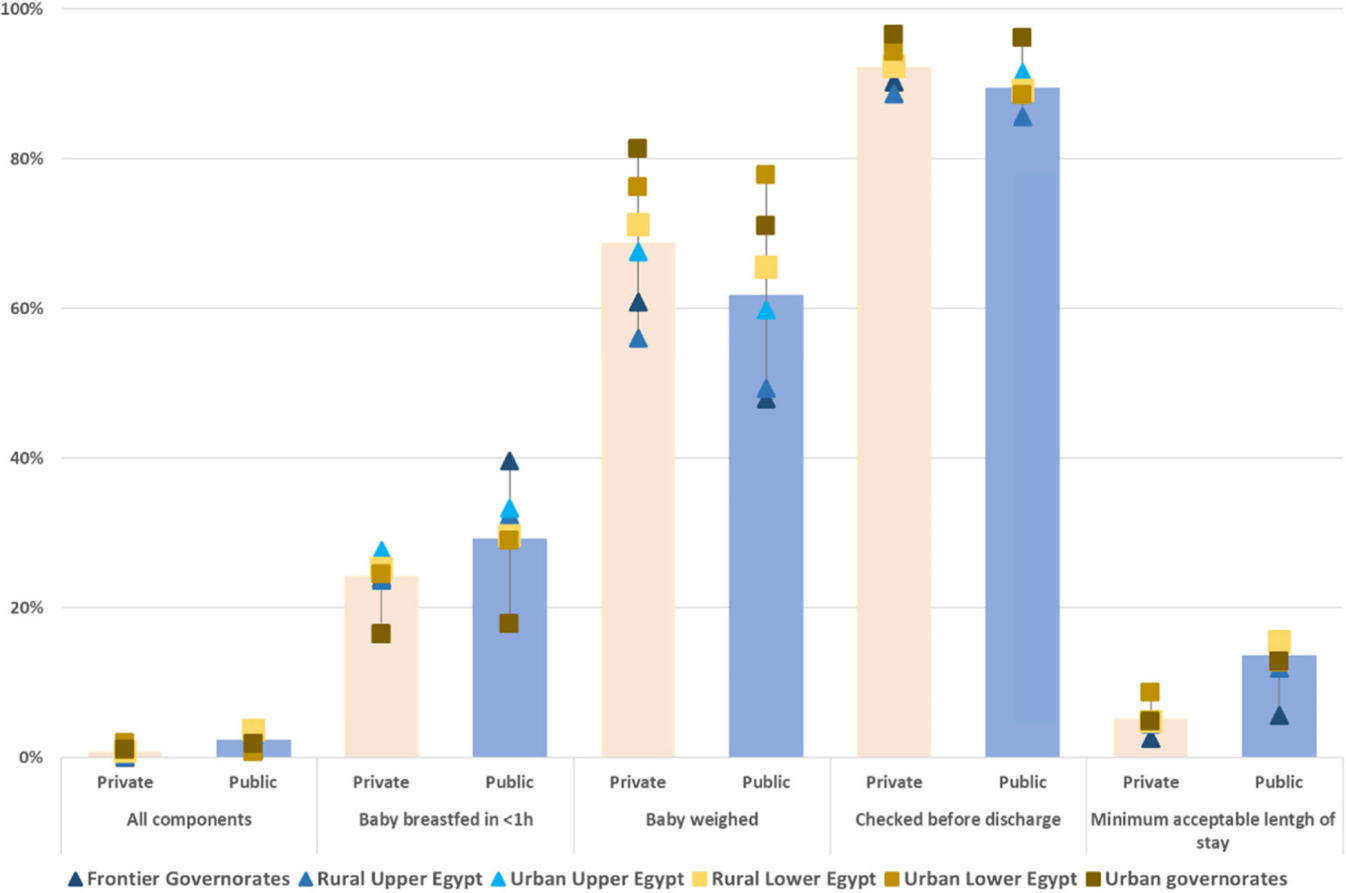
Percentage of women delivering in facilities reporting receiving each component of immediate postpartum care, and all components, by region

Across the six regions, having the baby weighed was the component with the largest absolute gap in provision, with 30 pp. between ULE and Frontier governorates for women delivering in public facilities and 25 pp. between frontier governorates and RUE in private (Fig. 5). This was followed by breastfeeding within 1 h among women in public facilities (22 pp. between frontier governorates and urban governorates).

## Discussion

Between 1991 and 1995 and 2010–2014, the percentage of women delivering in a health facility increased at the national level and in all regions. During this period, the private sector became the predominant provider of childbirth care, and the public sector declined in absolute terms. The private sector increase was observed across all regions and wealth groups. The private sector now assists more than half of the births in the country, ranging from 47% in Frontier governorates to 72% in urban Lower Egypt. The rise in the use of private facilities began among wealthier women first, followed by poorer women starting to use private providers between 2004 and 2008 and 2010–2014. On the other hand, in 2010–2014, 13% of women in need of childbirth care did not give birth in a facility and 8% were not attended by a SBA.

The main finding of this study is that, despite the large proportion of women delivering in health facilities, almost none received all four basic components of immediate postpartum care captured on the 2014 DHS. This was largely driven by three components: a very low percentage of women reporting a minimum acceptable length of stay, initiating breastfeeding within an hour of birth, and having their newborn weighed. The low provision of these care components used as proxies for quality of care likely shows a major quality gap that was present in both public and private sectors.

We identified an important proportion of women who did not access a facility birth or receive SBA-attended home-based childbirth care. Studies [25–27] in Egypt have highlighted that, in some regions, women living might still have difficulties accessing services. For instance due to long distances and the lack of night-time or alternative emergency services at the limits of public facilities’ catchment areas [25]. In Upper Egypt, some women reported distance, transportation and services’ costs as barriers to access antenatal and medical treatment services [26]. In the 2004–2008 DHS recall period, 63% of women delivering at home reported that giving birth in a facility was not necessary, while 23% highlighted concerns with the price of care [27]. Although free childbirth care should be available in public facilities in Egypt for those unable to pay for it, it has been observed that this policy failed to reach the poorest women, who end up delivering at home and do not necessarily benefit from these entitlements [17]. While public health insurance covers facility deliveries, poorer, less educated and rural women were less likely to be covered by health insurance [28].

Egypt is one of the LMICs with the highest share of women receiving childbirth care from private providers globally [24] and both socio-cultural and economic capital are strong determinants of facility-based childbirth care in Egypt [17]. However, we observed in our study that not only wealthy households but also the poorest have turned to private providers for childbirth care, particularly in the most recent period examined. The contribution of these services to families’ out-of-pocket health expenses is likely to be significant; the mean price of a birth (either vaginal or C-section) as measured on the 2008 DHS was four times higher in private compared to public facilities [17]. Given the substantial financial burden of such expenditure on families, the impact of the increase in private care utilisation among women from poorer households requires further research. Furthermore, the role of private providers potentially promoting unnecessary medical care must be assessed. Egypt is now one of the countries with the highest percentage of births by C-section [29]. The C-section rate has increased consistently and is likely influenced by the use of private care [30, 31].

The significant turn to private childbirth care despite its high price raises questions on Egyptians’ perceptions of public services compared to private providers. Physicians commonly work simultaneously in both public and private sectors (i.e., dual practice), and they may refer public patients to their private practice for certain services. A survey of physicians showed that 89% had more than one job, and 16% had three or more [32]. Furthermore, although private services are not necessarily more effective or efficient than public ones, they may provide more timely and hospitable services [33]. In parts of Lower and Upper Egypt, qualitative research identified significant deficiencies in the care received by women in public health services during pregnancy and childbirth. These included lack of explanations to patients, not obtaining consent for treatment, and not respecting their right to privacy and confidentiality. Further, some women complained of disrespectful care, which might be linked to lack of adequate training in doctor-patient communication and doctor’s limited time with patients [25]. This evidence, along with reports of high caseloads influencing doctors’ management of labour [34, 35] and deficits in staffing, distribution of workforce in rural public facilities [36] can be particularly relevant to understanding the shift to private care. In a nationally-representative survey, the three reasons most commonly cited as primary contributors to satisfaction across different services were perceived quality, good communication skills, and reasonable financial and physical access [37]. Egyptian women expect to deliver in a caring and respectful environment, conditions that may often clash with their experiences with health providers [38]. Therefore, poor patient-centred care may be a key factor behind women preferring private facilities to public ones. Indeed, our results showing that essential components of immediate postpartum care are as low in the private sector as in the public sector imply that the preferences for private care might be based on trust, communication, and respect rather than evidence-based care.

Despite the high percentage of women delivering in health facilities and with a SBA, it is unlikely that similar numbers are receiving this childbirth care with adequate quality. Our analysis of the components of immediate postpartum care revealed the limitation of relying on coverage indicators of facility and SBA-attended deliveries as proxies for the percentage of women receiving good quality care or evidence-based content of childbirth care. This finding is aligned with existing evidence and highlights the need to increase the focus on quality measures [3–6, 39]. In Egypt, the substandard provision of immediate postpartum care documented by our analysis concurs with previous evidence [34, 35, 40, 41] and raises questions about the quality of childbirth care provided in both public and private sector facilities. This is well-illustrated by the alarmingly low percentage of women staying for an acceptable amount of time after birth. Egypt is one of the LMICs with the shortest length of stay for both vaginal and C-section deliveries, and the shortest length of stay for singleton vaginal deliveries – only half a day [42]. Early discharge can have negative effects on both women and newborns. For instance, it was linked to higher neonatal readmission in an Egyptian hospital [43]. The short periods of stay after birth are of special concern considering the increase in the percentage of C-sections in Egypt—54% of all births in 2010–2014. For public services, previous research suggests that high caseloads may be causing bed shortages [35], which in some areas may be exacerbated by understaffing in facilities [36]. Previous research on C-sections in public hospitals suggests that unnecessary C-sections are driven by a combination of lack of training and supervision and doctors convenience incentives (i.e. doctors choosing the shortest delivery option, in which timing can also be decided) [44]. It is possible that these factors also influence the provision of postpartum care and is unclear whether they may influence private provision too. For private services, the percentage of women reporting an acceptable length of stay was consistently lower compared to public services for wealthier and poorer households, urban and rural, and vaginal and C-section deliveries. The reasons behind this inadequate care in private facilities must be identified and addressed by policymakers. For example, it would be important to understand what proportions of women reporting to have delivered with a private doctor (*tabib khas*) delivered in public facilities under the *fondoqy* scheme versus in private clinics. Second, the physical structure of private clinics needs to be explored further, including whether possibilities for a sufficient lentgh of postpartum stay exist (inpatient beds, overnight staffing and adequate nursing care, etc.).

The proportion of women reporting breastfeeding their baby within the first hour was remarkably low, despite evidence suggesting better breastfeeding outcomes may be more favourable in facility births compared to births at home [45]. Delivery by C-section has been observed to delay breastfeeding initiation in different settings [46–49]. In a recent report, UNICEF and WHO observed that early initiation rates were significantly lower in newborns delivered by C-section compared to vaginal delivery in 45 out of 51 LMICs studied, including Egypt [49]. Considering the large percentage of births by C-section in Egypt [30], it is particularly relevant that guidelines and interventions are targeted to improve early breastfeeding initiation after this type of delivery. One such intervention may be to introduce skin-to-skin contact after C-sections [50]. In addition, trained staff who are knowledgeable enough to facilitate skin-to-skin and inform and support mothers in the breastfeeding process, along with monitoring systems tracking improvement, can help support early initiation [49]. A small cross-sectional study with nursing students in Cairo observed weak breastfeeding knowledge [51], while previous evidence in the country suggests that training primary care providers to promote and support breastfeeding can improve the adequacy of postnatal counselling [52]. In our study, early breastfeeding was also more common in Frontier governorates and Upper Egypt and particularly low in Urban Governorates. These differences may be influenced by differences in the percentage of C-sections [22, 53], but also by the knowledge and preferences of mothers, providers and communities and existing initiatives supporting breastfeeding [53]. Some evidence has pointed to rural mothers being aware of the benefits of breastfeeding [54], and mothers in Lower Egypt delaying breastfeeding initiation to an hour after delivery more often than in Upper Egypt [55]. Future research should focus on analysing the effect of increased C-sections on breastfeeding and understand other barriers or enablers of early initiation of breastfeeding in Egypt.

In contrast to length of stay and breastfeeding initiation, most women delivering in a facility reported being checked while still in the facility before discharge. However, the DHS only asks women if someone either inquired about their health or examined them. This means that many of these women may have never received a physical or complete examination. Further research would be needed to understand to assess whether the percentage of women who actually received a physical examination according to guidelines before discharge really approximates what is reported. Similarly, the percentage of women reporting their baby weight measured was higher than length of stay and breastfeeding initiation. However, 30 to 40% of women still did not receive a fairly simple procedure, despite the importance of low birth weight as a known risk factor [56, 57]. The causes for providers not measuring birth weight routinely, specially in rural and poorer households, should be further explored.

When considering why components of immediate postpartum care were provided differently across sectors, it is important to bear in mind that different levels of enforcement, incentives and resources play a role. For instance, it has been highlighted that private neonatal clinics are hardly supervised or monitored by the MOHP [58]. Given that private providers attend most deliveries, it is critical that better regulation and monitoring are put in place. In public services, lack of adherence to guidelines, training, supportive supervision and coordinated referral systems have been documented, leaving health workers unsupported and unprepared to perform their jobs correctly [44, 59]. It is currently unclear whether private providers may experience these issues too; greater access to information from private facilities should be required for policy and research purposes. In addition, health workers’ move to the private sector for better salaries [36, 58, 60], leaving public facilities with less experienced staff and, sometimes, understaffed [36]. Urgent action is required to understand and address the drivers of poor quality in all its forms, including insufficient resources in the health system, poor compliance, perverse incentives, enforcement of existing standards, and providers’ behaviours moving between the private and public sector.

Immediate steps can be made towards improving the quality of childbirth care. For instance, midwives can have a greater role during and right after delivery, providing services (early breastfeeding, pre-discharge checks) that doctors may not have time for, and supporting women during delivery [30]. This task-shifting is likely to provide better patient-centred care to women and help with understaffing. Educational interventions targeting health providers and poor women in childbearing period could be an effective approach to make women more aware of the care they should be provided [61]. To better inform policymaking on a timely basis, routine data collection systems need to be developed to capture quality of care in key moments before, during and after childbirth. New specialised tools are being developed to understand how mothers are treated during childbirth and could be used to track the care mothers receive [62]. Moreover, further resources and attention need to be placed in developing systems that collect, harmonize and make health system data from both public and private providers accessible to policymakers and researchers [4].

### Limitations

This study benefited from comparable data collected in Egypt over a period of 23 years. The limitations of this study include the fact that surveys only captured live births, thus information about women experiencing negative outcomes (e.g., stillbirths, miscarriage, maternal death) is not reflected in this sample. In addition, some women have to recall care received up to 5 years ago and during which they may have received anaesthesia. These aspects may might also have affected the ability of some women to remember whether or how they received a specific intervention. We analysed care for most recent live births, and thus slightly underestimated the experiences of women who had more than one live birth in the recall period. Moreover, DHS surveys are cross-sectional and conducted every four to 5 years, making impossible to follow respondents over time.

Household surveys such as DHS and Multiple Indicator Cluster Surveys (MICS) do not capture receipt of key intrapartum care interventions such as use of uterotonics or monitoring of progress of labour [63]. Our ability to describe the content of childbirth was limited to the proxy components capturing only some aspects of clinical and process quality. As a result, important aspects such as interventions provided during delivery, the patient-provider interaction and respectful care could not be explored. However, the available four elements can likely provide important signals about the quality of intrapartum care and certainly reveal significant gaps in immediate postpartum care offered across the different categories of delivery systems. Given the systemic issues influencing quality of care in Egypt [58] and documented substandard intrapartum practices [34, 35], tools that can capture better measures of quality of care during and after delivery should be developed. However, such data and indicators are unlikely to be collected from women themselves, due to poor validity [64–67]. Finally, it was not possible to assess whether women in public hospitals were in a public or a paying/private section. The private sector includes a range of providers with different service quality and capacity to provide services adequately. Due to the nature of the questions in the survey, our analysis was not capable of differentiating between these providers to assess if critical differences exist between them. These challenges within DHS have been voiced elsewhere [68] and should considered in the design of future surveys looking into maternal health services.

## Conclusion

Between 1991 and 2014, Egypt experienced remarkable improvements in the percentage of women delivering in a facility and with an SBA across all regions and wealth groups. Crucially, during this period, the private sector became the main provider of childbirth care in the country. Studying the reasons behind this shift to private childbirth care can provide valuable information on dimensions of quality that may need improvement in public facilities, such as trust, communication and respect.

Despite the large percentage of women delivering in facilities, almost none of them reported received all the basic components of immediate postpartum care captured on the 2014 DHS, regardless of sector. This evidence suggests that most providers failed to provide childbirth care according to Egyptian or international guidelines. A combination of different factors is likely to influence substandard care, including insufficient staff, resources and training, lack of adherence to guidelines, inadequate supervision, and suboptimal incentives for provision of high-quality care. These factors need to be studied further to be addressed adequately. Researchers and policymakers must prioritize understanding the determinants of substandard childbirth care and developing policies and allocating resources to address them. Until these steps are taken, Egypt is likely to miss many of the benefits that their high levels of coverage of facility deliveries would be expected to confer on maternal and neonatal health.

## Supplementary information

**Additional file 1.** Indicators and definitions used [24, 42, 64–67, 69, 70].

**Additional file 2.** Characteristics of the study population.

**Additional file 3. **Percentages, 95% confidence intervals and *p*-values of the data used in Figs. 2, 3 and 4.

## Data Availability

The datasets generated and/or analysed during the current study are available in the Demographic and Health surveys repository, available at: https://dhsprogram.com/data/Using-Datasets-for-Analysis.cfm.

## Abbreviations

C-section: Caesarean section
DHS: Demographic and Health Survey
HDX: Humanitarian Data Exchange
HIO: Health insurance organization
LMICs: Low- and Middle-income countries
MICS: Multiple Indicator Cluster Surveys
MOHP: Ministry of Health and Population
NGOs: Non-governmental organizations
RLE: Rural Lower Egypt
RUE: Rural Upper Egypt
SBA: Skilled birth attendant
ULE: Urban Lower Egypt
UNICEF: United Nations International Children’s Emergency Fund
UUE: Urban Upper Egypt
WHO: World Health Organization
