# Supplementary material for: Childbirth care in Egypt: a repeat cross-sectional analysis using Demographic and Health Surveys between 1995 and 2014 examining use of care, provider mix and immediate postpartum care content
Source: BMC Pregnancy Childbirth. 2020 Jan 20;20:46. doi: 10.1186/s12884-020-2730-8 (PMC6971907; doi:10.1186/s12884-020-2730-8)
Supplement: Supplementary file 1 — Additional file 1. Indicators and definitions used [24, 42, 64–67, 69, 70]. [file 12884_2020_2730_MOESM1_ESM.docx]

### Additional file 1: Indicators and definitions used

#### Women in need of childbirth care, location of delivery and delivery attendant

Women were considered to need childbirth care if they had a live birth in the survey’s recall period [24]. Women were asked where they gave birth and who assisted them with the delivery. Women reporting to have given birth in any health facility were considered to have had a facility delivery and women reporting to have delivered in a domestic setting were considered to have had a home delivery. Women were considered to have been assisted by an SBA if they reported that a doctor, nurse, or midwife assisted them during childbirth. Traditional birth attendants (in Arabic *daya,* sing*.*) were not considered to be SBAs. Women not knowing or having a missing value for birth location (1.0% of the pooled dataset of five surveys) or attendant (1.7%) were excluded from the analysis.

#### Sector of childbirth care provision (public or private)

Women delivering in facilities were categorised into public or private-sector users. Public-sector childbirth facilities included urban hospitals, urban health units, health offices, rural hospitals, rural health units, maternal and child health centres, and the response “other government facilities”. Private-sector providers included non-governmental organizations, private hospitals (*mustashfa khas*), private clinics *(ayada khasa*), and private doctors (*tabib khas*).

#### Household Wealth

We characterized the socio-economic status of women receiving childbirth care using household wealth quintiles. Quintiles were created measuring household asset ownership and creating appropriate thresholds [69,70]. To avoid the use of subpopulations with small samples (n<100), we combined quintiles one and two to represent the poorest 40% of households, and quintiles 4 and 5 to represent the wealthiest 40%.

#### Components of immediate postpartum care on 2014 survey

The DHS did not ask women to report on content, components or quality of intrapartum care (beyond the cadre of the birth attendant and whether the birth was by a C-section) likely because of the limited validity of these indicators [64–67]. Thus, to assess the content of childbirth care, we used elements of immediate postpartum care as proxies instead.

We examined content of immediate postpartum care reported by women delivering in health facilities on the 2014 survey using four components asking whether or not: 1) women initiated breastfeeding immediately (within an hour of birth), 2) the baby was weighed, 3) the woman was checked (i.e., someone checked on her health, although the question did not specify a physical examination) while still in the facility, and 4) women reported a minimally acceptable length-of stay in the facility. We defined a minimum acceptable length of stay as being 24 hours following a vaginal delivery and 72 hours after a C-section [42]. We assessed the percentage of women receiving each component as well as the percentage receiving all four components. Women responding “don’t know” or having a missing value for any component were classified as not receiving such component. In our analysis sample, missing answers amounted to 4.7% of women reporting on initiation of breastfeeding, 0.06% reporting on a check before discharge, and none of the women reporting on length-of-stay or on the baby being weighed.
