# Supplementary material for: Childbirth care in Egypt: a repeat cross-sectional analysis using Demographic and Health Surveys between 1995 and 2014 examining use of care, provider mix and immediate postpartum care content
Source: BMC Pregnancy Childbirth. 2020 Jan 20;20:46. doi: 10.1186/s12884-020-2730-8 (PMC6971907; doi:10.1186/s12884-020-2730-8)
Supplement: Supplementary file 2 — Additional file 2. Characteristics of the study population. [file 12884_2020_2730_MOESM2_ESM.docx]

|  | **1991-1995** | | **1996-2000** | | **2001-2005** | | **2004-2008** | | **2010-2014** | | **Total** | |
| --- | --- | --- | --- | --- | --- | --- | --- | --- | --- | --- | --- | --- |
|  | **Weighed %** | **N** | **Weighed %** | **N** | **Weighed %** | **N** | **Weighed %** | **N** | **Weighed %** | **N** | **Weighed %** | **N** |
|  |  |  |  |  |  |  |  |  |  |  |  |  |
| **In need of delivery care** | 100% | 7,964 | 100% | 7,987 | 100% | 9,933 | 100% | 8,022 | 100% | 11,481 | 100% | 45,387 |
| **Received facility-based care** | 35% | 2,631 | 51% | 4,101 | 66% | 6,384 | 72% | 5,713 | 87% | 10,139 | 65% | 28,968 |
| **Type of delivery** |  |  |  |  |  |  |  |  |  |  |  |  |
| Vaginal | 93% | 7,469 | 89% | 7,088 | 79% | 7,926 | 71% | 5,781 | 46% | 5,328 | 73% | 33,592 |
| C-section | 7% | 495 | 11% | 899 | 21% | 2,007 | 29% | 2,241 | 54% | 6,153 | 27% | 11,795 |
| **Age** |  |  |  |  |  |  |  |  |  |  |  |  |
| 15-19 | 4% | 336 | 4% | 285 | 4% | 389 | 3% | 275 | 3% | 321 | 4% | 1,606 |
| 20-29 | 51% | 3933 | 52% | 4090 | 55% | 5392 | 58% | 4566 | 55% | 6226 | 54% | 24,207 |
| 30-39 | 36% | 2980 | 37% | 3012 | 35% | 3474 | 33% | 2684 | 37% | 4313 | 36% | 16,463 |
| 40-49 | 9% | 715 | 7% | 600 | 7% | 678 | 6% | 497 | 5% | 621 | 7% | 3,111 |
| **Education** |  |  |  |  |  |  |  |  |  |  |  |  |
| No education | 44% | 3,693 | 39% | 3,081 | 30% | 3,189 | 25% | 2,183 | 18% | 1,927 | 30% | 14,073 |
| Some primary | 22% | 1,647 | 16% | 1,229 | 13% | 1,261 | 10% | 819 | 9% | 982 | 13% | 5,938 |
| Primary complete / some secondary | 29% | 2,212 | 37% | 3,005 | 46% | 4,537 | 51% | 4,048 | 57% | 6,653 | 45% | 20,455 |
| Secondary complete / higher | 6% | 412 | 9% | 672 | 11% | 946 | 13% | 972 | 16% | 1,919 | 11% | 4,921 |
| **Marital status** |  |  |  |  |  |  |  |  |  |  |  |  |
| Married | 98% | 7,806 | 98% | 7,847 | 98% | 9,727 | 98% | 7,880 | 98% | 11,267 | 98% | 44,527 |
| Separated, Divorced, Widowed or not living together | 2% | 158 | 2% | 140 | 2% | 206 | 2% | 142 | 2% | 214 | 2% | 860 |
| **Type of residence** |  |  |  |  |  |  |  |  |  |  |  |  |
| Rural | 59% | 4976 | 60% | 4601 | 62% | 6145 | 62% | 5005 | 68% | 6716 | 63% | 27443 |
| Urban | 41% | 2988 | 40% | 3386 | 38% | 3788 | 38% | 3017 | 32% | 4765 | 37% | 17944 |
| **Household wealth** |  |  |  |  |  |  |  |  |  |  |  |  |
| 1 | 22% | 2000 | 20% | 1599 | 20% | 2315 | 19% | 1764 | 17% | 2063 | 19% | 9741 |
| 2 | 20% | 1631 | 19% | 1448 | 20% | 2083 | 20% | 1637 | 19% | 2105 | 20% | 8904 |
| 3 | 21% | 1565 | 20% | 1631 | 22% | 1962 | 21% | 1665 | 25% | 2344 | 22% | 9167 |
| 4 | 19% | 1410 | 22% | 1711 | 21% | 1909 | 21% | 1514 | 21% | 2428 | 21% | 8972 |
| 5 | 19% | 1358 | 19% | 1598 | 18% | 1664 | 19% | 1442 | 17% | 2541 | 18% | 8603 |
| **Region of residence** |  |  |  |  |  |  |  |  |  |  |  |  |
| Urban governorates | 19% | 1124 | 17% | 1378 | 15% | 1530 | 16% | 1074 | 11% | 1732 | 15% | 6838 |
| Urban Lower Egypt | 10% | 640 | 12% | 856 | 10% | 708 | 10% | 711 | 9% | 1173 | 10% | 4088 |
| Rural Lower Egypt | 29% | 1661 | 31% | 2148 | 31% | 2161 | 34% | 2275 | 39% | 3135 | 33% | 11380 |
| Urban Upper Egypt | 11% | 827 | 11% | 826 | 13% | 1263 | 11% | 945 | 11% | 1381 | 11% | 5242 |
| Rural Upper Egypt | 29% | 3016 | 28% | 2239 | 30% | 3786 | 27% | 2560 | 29% | 3330 | 29% | 14931 |
| Frontier Governorates | 1% | 696 | 2% | 540 | 1% | 485 | 1% | 457 | 1% | 730 | 1% | 2908 |
|  |  |  |  |  |  |  |  |  |  |  |  |  |
