# Supplementary material for: Childbirth care in Egypt: a repeat cross-sectional analysis using Demographic and Health Surveys between 1995 and 2014 examining use of care, provider mix and immediate postpartum care content
Source: BMC Pregnancy Childbirth. 2020 Jan 20;20:46. doi: 10.1186/s12884-020-2730-8 (PMC6971907; doi:10.1186/s12884-020-2730-8)
Supplement: Supplementary file 3 — Additional file 3. Percentages, 95% confidence intervals and p-values of the data used in Figs. 2, 3 and 4. [file 12884_2020_2730_MOESM3_ESM.docx]

|  | **1991-1995** | | **1996-2000** | | **2001-2005** | | **2004-2008** | | **2010-2014** | | **P-value** |  |
| --- | --- | --- | --- | --- | --- | --- | --- | --- | --- | --- | --- | --- |
|  | **%** | **95% CI** | **%** | **95% CI** | **%** | **95% CI** | **%** | **95% CI** | **%** | **95% CI** |  |  |
|  |  |  |  |  |  |  |  |  |  |  |  |  |
| **Home delivery** | 65% | (0.62-0.68) | 49% | (0.47-0.52) | 34% | (0.31-0.36) | 28% | (0.26-0.29) | 13% | (0.11-0.14) | <0.001 |  |
| **Facility delivery** | 35% | (0.32-0.38) | 51% | (0.48-0.53) | 66% | (0.64-0.69) | 72% | (0.71-0.74) | 87% | (0.86-0.89) |  |  |
|  |  |  |  |  |  |  |  |  |  |  |  |  |
| **Facility delivery: Public** | 19% | (0.17-0.21) | 23% | (0.21-0.25) | 26% | (0.24-0.28) | 27% | (0.25-0.28) | 25% | (0.23-0.26) | <0.001 |  |
| **Facility delivery: Private** | 16% | (0.14-0.18) | 28% | (0.25-0.30) | 41% | (0.25-0.30) | 46% | (0.44-0.47) | 63% | (0.61-0.65) |  |  |
|  |  |  |  |  |  |  |  |  |  |  |  |  |
| **Deliveries with skilled birth attendant (SBA)** | 49% | (0.46-0.52) | 63% | (0.61-0.66) | 76% | (0.74-0.78) | 80% | (0.78-0.81) | 92% | (0.91-0.93) | <0.001 |  |
|  |  |  |  |  |  |  |  |  |  |  |  |  |
| **Urban Governorates** |  |  |  |  |  |  |  |  |  |  |  |  |
| *Home delivery, no SBA* | 29% | (0.21-0.38) | 14% | (0.10-0.19) | 7% | (0.05-0.09) | 7% | (0.05-0.10) | 2% | (0.01-0.04) | <0.001 |  |
| *Home delivery, SBA* | 8% | (0.06-0.11) | 7% | (0.06-0.09) | 5% | (0.04-0.06) | 3% | (0.02-0.04) | 2% | (0.01-0.04) |  |  |
| *Public facility* | 38% | (0.32-0.44) | 41% | (0.37-0.46) | 44% | (0.39-0.48) | 40% | (0.36-0.44) | 36% | (0.31-0.42) |  |  |
| *Private facility* | 25% | (0.18-0.33) | 37% | (0.32-0.43) | 45% | (0.40-0.49) | 50% | (0.46-0.54) | 59% | (0.54-0.65) |  |  |
| **Urban Lower Egypt** |  |  |  |  |  |  |  |  |  |  |  |  |
| *Home delivery, no SBA* | 23% | (0.16-0.31) | 15% | (0.12-0.19) | 8% | (0.05-0.11) | 8% | (0.06-0.11) | 2% | (0.01-0.03) | <0.001 |  |
| *Home delivery, SBA* | 15% | (0.11-0.20) | 12% | (0.09-0.16) | 5% | (0.04-0.08) | 5% | (0.04-0.08) | 2% | (0.01-0.03) |  |  |
| *Public facility* | 25% | (0.21-0.31) | 25% | (0.20-0.30) | 25% | (0.20-0.31) | 25% | (0.21-0.29) | 25% | (0.20-0.30) |  |  |
| *Private facility* | 37% | (0.29-0.45) | 48% | (0.41-0.55) | 62% | (0.55-0.69) | 62% | (0.56-0.67) | 72% | (0.67-0.77) |  |  |
| **Rural Lower Egypt** |  |  |  |  |  |  |  |  |  |  |  |  |
| *Home delivery, no SBA* | 55% | (0.51-0.59) | 41% | (0.37-0.45) | 21% | (0.18-0.26) | 17% | (0.14-0.19) | 6% | (0.04-0.07) | <0.001 |  |
| *Home delivery, SBA* | 20% | (0.17-0.24) | 14% | (0.12-0.17) | 10% | (0.08-0.13) | 9% | (0.07-0.10) | 4% | (0.03-0.06) |  |  |
| *Public facility* | 13% | (0.10-0.15) | 16% | (0.14-0.18) | 22% | (0.19-0.26) | 22% | (0.20-0.24) | 19% | (0.17-0.21) |  |  |
| *Private facility* | 12% | (0.10-0.15) | 29% | (0.26-0.33) | 46% | (0.42-0.51) | 53% | (0.50-0.56) | 71% | (0.68-0.74) |  |  |
| **Urban Upper Egypt** |  |  |  |  |  |  |  |  |  |  |  |  |
| *Home delivery, no SBA* | 38% | (0.30-0.47) | 23% | (0.18-0.29) | 14% | (0.11-0.18) | 13% | (0.10-0.17) | 5% | (0.04-0.07) | <0.001 |  |
| *Home delivery, SBA* | 15% | (0.11-0.20) | 15% | (0.11-0.21) | 10% | (0.08-0.12) | 7% | (0.05-0.09) | 4% | (0.03-0.05) |  |  |
| *Public facility* | 26% | (0.20-0.32) | 33% | (0.27-0.40) | 29% | (0.25-0.34) | 31% | (0.27-0.36) | 30% | (0.27-0.34) |  |  |
| *Private facility* | 20% | (0.15-0.27) | 29% | (0.23-0.36) | 47% | (0.42-0.52) | 49% | (0.44-0.54) | 61% | (0.57-0.64) |  |  |
| **Rural Upper Egypt** |  |  |  |  |  |  |  |  |  |  |  |  |
| *Home delivery, no SBA* | 75% | (0.72-0.79) | 60% | (0.56-0.64) | 43% | (0.40-0.46) | 39% | (0.36-0.43) | 16% | (0.14-0.19) | <0.001 |  |
| *Home delivery, SBA* | 10% | (0.08-0.12) | 13% | (0.10-0.16) | 14% | (0.12-0.17) | 10% | (0.09-0.12) | 7% | (0.06-0.09) |  |  |
| *Public facility* | 9% | (0.08-0.11) | 15% | (0.13-0.18) | 19% | (0.17-0.22) | 23% | (0.21-0.26) | 25% | (0.22-0.29) |  |  |
| *Private facility* | 5% | (0.04-0.07) | 12% | (0.10-0.14) | 23% | (0.20-0.26) | 27% | (0.25-0.30) | 51% | (0.48-0.55) |  |  |
| **Frontier Governorates** |  |  |  |  |  |  |  |  |  |  |  |  |
| *Home delivery, no SBA* | 38% | (0.28-0.49) | 37% | (0.25-0.50) | 27% | (0.18-0.37) | 19% | (0.14-0.25) | 9% | (0.05-0.16) | <0.001 |  |
| *Home delivery, SBA* | 29% | (0.22-0.37) | 20% | (0.13-0.30) | 12% | (0.08-0.19) | 7% | (0.04-0.10) | 5% | (0.03-0.08) |  |  |
| *Public facility* | 21% | (0.16-0.27) | 27% | (0.21-0.34) | 35% | (0.30-0.40) | 43% | (0.37-0.49) | 39% | (0.30-0.49) |  |  |
| *Private facility* | 12% | (0.08-0.18) | 16% | (0.11-0.23) | 27% | (0.20-0.35) | 32% | (0.26-0.38) | 47% | (0.34-0.60) |  |  |
| **National** |  |  |  |  |  |  |  |  |  |  |  |  |
| *Home delivery, no SBA* | 51% | (0.48-0.54) | 37% | (0.34-0.39) | 24% | (0.22-0.26) | 20% | (0.19-0.22) | 8% | (0.07-0.09) | <0.001 |  |
| *Home delivery, SBA* | 14% | (0.12-0.16) | 12% | (0.11-0.14) | 10% | (0.09-0.11) | 8% | (0.07-0.08) | 5% | (0.04-0.05) |  |  |
| *Public facility* | 19% | (0.17-0.21) | 23% | (0.21-0.25) | 26% | (0.24-0.28) | 27% | (0.25-0.28) | 25% | (0.23-0.26) |  |  |
| *Private facility* | 16% | (0.14-0.18) | 28% | (0.25-0.30) | 41% | (0.38-0.43) | 46% | (0.44-0.47) | 63% | (0.61-0.65) |  |  |
|  |  |  |  |  |  |  |  |  |  |  |  |  |
| **Wealth quintiles 1 & 2** |  |  |  |  |  |  |  |  |  |  |  |  |
| *Home delivery,* | 84% | (0.82-0.86) | 72% | (0.69-0.74) | 52% | (0.49-0.55) | 46% | (0.44-0.49) | 22% | (0.20-0.24) | <0.001 |  |
| *Public facility* | 10% | (0.08-0.11) | 15% | (0.13-0.17) | 22% | (0.20-0.24) | 24% | (0.22-0.26) | 25% | (0.22-0.27) |  |  |
| *Private facility* | 6% | (0.11-0.15) | 13% | (0.11-0.15) | 26% | (0.23-0.28) | 29% | (0.27-0.32) | 54% | (0.51-0.57) |  |  |
| **Wealth quintiles 4 & 5** |  |  |  |  |  |  |  |  |  |  |  |  |
| *Home delivery,* | 41% | (0.37-0.45) | 27% | (0.24-0.29) | 15% | (0.14-0.17) | 11% | (0.09-0.12) | 5% | (0.04-0.06) | <0.001 |  |
| *Public facility* | 29% | (0.26-0.32) | 31% | (0.28-0.33) | 28% | (0.26-0.31) | 28% | (0.26-0.30) | 27% | (0.24-0.29) |  |  |
| *Private facility* | 43% | (0.39-0.46) | 43% | (0.39-0.46) | 57% | (0.54-0.59) | 61% | (0.59-0.64) | 69% | (0.66-0.71) |  |  |

|  | **All four components** | | **P-value** | **Baby breastfed in <1h** | | **P-value** | **Baby weighed** | | **P-value** | **Mother checked before discharge** | | **P-value** | **Minimum acceptable lentgh of stay** | | **P-value** |
| --- | --- | --- | --- | --- | --- | --- | --- | --- | --- | --- | --- | --- | --- | --- | --- |
|  | **%** | **95% CI** |  | **%** | **95% CI** |  | **%** | **95% CI** |  | **%** | **95% CI** |  | **%** | **95% CI** |  |
| **Private sector** |  |  |  |  |  |  |  |  |  |  |  |  |  |  |  |
| *Wealth quintiles 1 & 2* | 1% | (0.00-0.01) | 0.551 | 23% | (0.21-0.26) | 0.029 | 59% | (0.56-0.62) | <0.001 | 89% | (0.87-0.91) | <0.001 | 5% | (0.04-0.07) | 0.057 |
| *Wealth quintile 3* | 0% | (0.00-0.01) |  | 27% | (0.24-0.31) |  | 69% | (0.66-0.72) |  | 91% | (0.90-0.93) |  | 4% | (0.03-0.05) |  |
| *Wealth quintiles 4&5* | 1% | (0.01-0.02) |  | 23% | (0.21-0.25) |  | 75% | (0.73-0.77) |  | 95% | (0.94-0.96) |  | 6% | (0.05-0.07) |  |
|  |  |  |  |  |  |  |  |  |  |  |  |  |  |  |  |
| *Urban Governorates* | 1% | (0.00-0.02) | 0.014 | 16% | (0.13-0.21) | 0.006 | 81% | (0.76-0.86) | <0.001 | 96% | (0.94-0.98) | 0.014 | 5% | (0.03-0.07) | 0.003 |
| *Urban Lower Egypt* | 2% | (0.01-0.04) |  | 24% | (0.21-0.28) |  | 76% | (0.72-0.80) |  | 94% | (0.92-0.96) |  | 9% | (0.07-0.11) |  |
| *Rural Lower Egypt* | 1% | (0.00-0.01) |  | 25% | (0.23-0.28) |  | 71% | (0.69-0.74) |  | 92% | (0.91-0.94) |  | 5% | (0.04-0.06) |  |
| *Urban Upper Egypt* | 1% | (0.00-0.02) |  | 28% | (0.24-0.32) |  | 68% | (0.63-0.71) |  | 93% | (0.91-0.95) |  | 5% | (0.04-0.07) |  |
| *Rural Upper Egypt* | 0% | (0.00-0.00) |  | 24% | (0.21-0.27) |  | 56% | (0.52-0.60) |  | 89% | (0.87-0.91) |  | 5% | (0.03-0.06) |  |
| *Frontier Governorates* | 0% | (0.00-0.00) |  | 24% | (0.18-0.32) |  | 61% | (0.54-0.68) |  | 90% | (0.87-0.93) |  | 3% | (0.01-0.05) |  |
|  |  |  |  |  |  |  |  |  |  |  |  |  |  |  |  |
| **Public sector** |  |  |  |  |  |  |  |  |  |  |  |  |  |  |  |
| *Wealth quintiles 1 & 2* | 98% | (0.96-0.99) | 0.622 | 70% | (0.67-0.74) | 0.013 | 48% | (0.44-0.52) | <0.001 | 15% | (0.13-0.18) | <0.001 | 84% | (0.82-0.87) | 0.181 |
| *Wealth quintile 3* | 97% | (0.95-0.98) |  | 65% | (0.59-0.71) |  | 40% | (0.35-0.45) |  | 9% | (0.07-0.12) |  | 88% | (0.84-0.91) |  |
| *Wealth quintiles 4&5* | 98% | (0.97-0.99) |  | 74% | (0.70-0.78) |  | 29% | (0.26-0.33) |  | 7% | (0.06-0.09) |  | 87% | (0.85-0.90) |  |
|  |  |  |  |  |  |  |  |  |  |  |  |  |  |  |  |
| *Urban Governorates* | 2% | (0.01-0.04) | 0.025 | 18% | (0.13-0.25) | 0.003 | 71% | (0.63-0.78) | <0.001 | 96% | (0.93-0.98) | 0.025 | 13% | (0.09-0.17) | 0.317 |
| *Urban Lower Egypt* | 1% | (0.00-0.03) |  | 29% | (0.22-0.37) |  | 78% | (0.72-0.83) |  | 88% | (0.84-0.92) |  | 13% | (0.09-0.18) |  |
| *Rural Lower Egypt* | 4% | (0.02-0.06) |  | 30% | (0.25-0.35) |  | 65% | (0.60-0.70) |  | 89% | (0.86-0.92) |  | 15% | (0.12-0.20) |  |
| *Urban Upper Egypt* | 3% | (0.01-0.06) |  | 33% | (0.28-0.40) |  | 60% | (0.54-0.65) |  | 91% | (0.88-0.94) |  | 15% | (0.12-0.20) |  |
| *Rural Upper Egypt* | 1% | (0.01-0.03) |  | 32% | (0.28-0.37) |  | 49% | (0.45-0.53) |  | 86% | (0.82-0.89) |  | 12% | (0.09-0.15) |  |
| *Frontier Governorates* | 1% | (0.00-0.04) |  | 40% | (0.29-0.52) |  | 48% | (0.41-0.55) |  | 91% | (0.84-0.95) |  | 6% | (0.03-0.10) |  |
